# Liver morphometrics and metabolic blood profile across divergent phenotypes for feed efficiency in the bovine

**DOI:** 10.1186/s13028-017-0292-1

**Published:** 2017-04-26

**Authors:** Yuri Regis Montanholi, Livia Sadocco Haas, Kendall Carl Swanson, Brenda Lee Coomber, Shigeto Yamashiro, Stephen Paul Miller

**Affiliations:** 1 Department of Animal Science and Aquaculture, Faculty of Agriculture, Dalhousie University, 58 River Road, Bible Hill, Truro, NS B2N 5E3 Canada; 2 Faculdade de Medicina Veterinária, Universidade Federal do Rio Grande do Sul, Porto Alegre, RS 91540-000 Brazil; 3 Department of Animal Sciences, North Dakota State University, Fargo, ND 58102 USA; 4 Department of Biomedical Sciences, University of Guelph, Guelph, ON N1G 2W1 Canada; 5 Department of Animal Biosciences, University of Guelph, Guelph, ON N1G 2W1 Canada; 6 Angus Genetics Inc, Saint Joseph, MO 64506 USA

**Keywords:** Albumin, Carbon dioxide, Cholesterol, Creatinine, Energy metabolism, Hepatocyte dimensions, Histomorphometry, Liver size, Metabolic rate, Residual feed intake

## Abstract

**Background:**

Feed costs are a major expense in the production of beef cattle. Individual variation in the efficiency of feed utilization may be evident through feed efficiency-related phenotypes such as those related to major energetic sinks. Our objectives were to assess the relationships between feed efficiency with liver morphometry and metabolic blood profile in feedlot beef cattle.

**Methods:**

Two populations (A = 112 and B = 45) of steers were tested for feed efficiency. Blood from the 12 most (efficient) and 12 least feed inefficient (inefficient) steers from population A was sampled hourly over the circadian period. Blood plasma samples were submitted for analysis on albumin, aspartate aminotransferase, γ-glutamyl transpeptidase urea, cholesterol, creatinine, alkaline phosphatase, creatine kinase, lipase, carbon dioxide, β-hydroxybutyrate, acetate and bile acids. Liver tissue was also harvested from 24 steers that were blood sampled from population A and the 10 steers with divergent feed efficiency in each tail of population B was sampled for microscopy at slaughter. Photomicroscopy images were taken using the portal triad and central vein as landmarks. Histological quantifications included cross-sectional hepatocyte perimeter and area, hepatocyte nuclear area and nuclei area as proportion of the hepatocyte area. The least square means comparison between efficient and inefficient steers for productive performance and liver morphometry and for blood analytes data were analyzed using general linear model and mixed model procedures of SAS, respectively.

**Results:**

No differences were observed for liver weight; however, efficient steers had larger hepatocyte (i.e. hepatocyte area at the porta triad 323.31 vs. 286.37 µm^2^) and nuclei dimensions at portal triad and central vein regions, compared with inefficient steers. The metabolic profile indicated efficient steers had lower albumin (36.18 vs. 37.65 g/l) and cholesterol (2.62 vs. 3.05 mmol/l) and higher creatinine (118.59 vs. 110.50 mmol/l) and carbon dioxide (24.36 vs. 23.65 mmol/l) than inefficient steers.

**Conclusions:**

Improved feed efficiency is associated with increased metabolism by the liver (enlarged hepatocytes and no difference on organ size), muscle (higher creatinine) and whole body (higher carbon dioxide); additionally, efficient steers had reduced bloodstream pools of albumin and cholesterol. These metabolic discrepancies between feed efficient and inefficient cattle may be determinants of productive performance.

## Background

Feed costs are a major expense in the production of beef cattle [1]. An avenue for decreasing feeding costs is the improvement of feed utilization through utilization of cattle with improved feed efficiency. The identification of such cattle is limited by practical phenotypes for feed efficiency with application in commercial herds. The evaluation of physiological aspects underlying feed efficiency has been studied in beef cattle through residual feed intake (RFI) [2, 3]. This feed efficiency measure reflects the variation in feed intake upon adjustment for body size, body weight gain and body composition. Thus, the residual of this determination represents variation in the requirements for basal metabolic processes rather than differences in productivity, constituting a relevant trait in the search of biological indicators for feed efficiency.

Requirements for basal metabolic processes represent a large amount of total energy expenditure of the animal [4]. Cattle raised under the same conditions, at the same physiological state, and with similar genetic composition vary in basal energy requirements [5, 6]. A significant portion of these energy requirements supports the metabolism of visceral organs [7]. Despite the fact that these organs only represent approximately 6–10% of body-weight, about 40–50% of the total basal energy requirements is due to the metabolism of the liver and digestive tract [8]. Liver metabolism accounts for about half of this amount [9], while comprising 1.45% of body weight in beef steers [10].

Variation in feed intake results in changes to the metabolic rate of visceral organs [11], which in turn creates fluctuations in blood flow affecting both organ size and tissue metabolic activity [12]. Johnson et al. [7] found that an increase in the functional workload of the liver through dietary manipulation in cattle and sheep, resulted in an increase in liver weight, which is associated with hepatocyte hypertrophy [13]. Conversely, Zaitoun et al. [14] observed that a decrease in liver metabolism through surgical manipulation in rats resulted in hepatocyte hypotrophy. Interestingly, Montanholi et al. [15] observed an increase in the small intestine crypt cellularity in cattle with improved feed efficiency. Such histological and functional evidence is associated with increased metabolic rate of the digestive tract in snakes [16] and also associated with variation in feed efficiency in beef cattle calves [17].

Besides micro-structural changes, variation in workload of the liver and other metabolic systems is also reflected in blood plasma analytes; such findings were reported by Gonano et al. [18] while evaluating a series of blood analytes over the circadian period and across physiological states in beef heifers; by Bourgon et al. [19] while evaluating blood analytes over the ultradian period in young beef bulls and; by Richardson et al. [20] while evaluating blood analytes over spot sampling in feedlot cattle. Products related to liver function including albumin, cholesterol, urea and bile acids (BA); enzymes related to liver function including alkaline phosphatase (ALP), aspartate aminotransferase (AST), γ-glutamyl transpeptidase (GGT) and lipase; indicators of energetic status including acetate, β-hydroxybutyrate (BHBA) and carbon dioxide (CO_2_); and indicators of muscle metabolism including creatinine and creatine kinase (CK) are important analytes to be evaluated over the circadian period due to their relevance in the basal energy requirements [20, 21].

Since variation in feed efficiency results in differences in feed intake and, consequently, impacts the workload placed on visceral organs in which metabolic changes affect tissue microarchitecture and blood metabolic profile, it can be hypothesized that these biological indicators may also indicate variation in feed efficiency. Therefore, our objectives were to characterize: (1) the morphometry of liver tissue; and (2) the circadian metabolic blood profile in beef cattle with divergent feed efficiency.

## Methods

### Experimental units and animal husbandry

The experiment followed recommendations outlined by the Canadian Council of Animal Care guidelines (2009). A total of 112 (population A) plus 45 (population B) crossbred steers with known dates of birth were fed for 140 days at the Elora Beef Research Centre, Canada. The breed composition of the steers was primarily 57.1% Angus, 29.6% Simmental and 3.5% Hereford for population A, and 33.0% Angus, 27.7% Charolais, and 13.9% Piedmontese for population B. The remaining breed composition was comprised of other European breeds in both populations. Steers were allowed to adjust to the facilities, feed and feeding system for 15 days prior to the start of the feeding and performance evaluation trial. Pens were naturally ventilated, bedded with wood shavings and each held 14–15 steers. Every pen contained four electronic feeding stations (Insentec, B.V., Marknesse, The Netherlands) with access to fresh water. Radio frequency identification tags (Allflex, St. Hyacinthe, Canada) were placed in the right ear of each steer to continuously record individual feeding events. Both populations were fed ad libitum a high moisture, corn-based diet similar to a formulation used elsewhere [23]. The ration was added with 28 mg of monensin (Rumensin^®^; Elanco, Greenfield, USA) per kilogram of dry matter, which is typical for commercial operations in regions that produce corn in North America. Every 28 days for a period of 140 days, in the morning, steers were weighed using a calibrated livestock scale and ultrasound scanned for body composition by a trained technician. Backfat thickness (BKFT; mm) and rib eye area (RBEA; cm^2^) were assessed using real-time ultrasound as described by [3]. Shortly after the end of the productive performance evaluation (10 ± 2.4 days), steers from population A were subjected to blood sampling and then sent to slaughter, steers from population B were sent to slaughter within 2 weeks after the completion of the performance evaluation. The average age of the steers at slaughter was 477 ± 21 days (mean ± standard deviation) for population A and 411 ± 18 days for population B. Both groups of steers were also weighed on the day before and on the day of the slaughter at the research station.

### Feed efficiency determination and feed efficiency ranking

Body weight (BW), body composition and feed intake measures were assessed to determine the residual feed intake following the methodology described by Montanholi et al. [3]. Briefly, the individual daily dry matter intake (DMI; kg/day) was computed by combining feeding events within each day. The daily feed intakes were filtered for outliers, which represented less than 2% of the feeding records, and converted to a dry matter basis. Average BW, BKFT and RBEA was calculated by computing the animals’ intercept plus the average daily gains of each of these traits times 70. Individual DMI, average BW gain, average BW and ultrasound measurements were used to calculate RFI. The most appropriate model for the population A had a R^2^ of 0.63, as presented by Montanholi et al. [24]. Similarly, for population B the best fit model had a R^2^ of 0.72, as computed by Montanholi et al. [23]. Steers were then ranked based on residual feed intake and the animals in the extremes for feed efficiency (12 efficient and 12 inefficient) were selected for circadian blood plasma metabolic profile analysis from population A only. In the case of biometry, liver morphometry and productive performance traits, steers from both populations were considered. Thus, the same 12 efficient and 12 inefficient steers from population A, subjected to blood sampling, were added to the 10 efficient and 10 inefficient steers from population B in these datasets based on statistics detailed below.

### Blood collection and processing

Steers from population A were blood sampled in six groups of four animals at 6 ± 3.6 days prior to slaughter. The methodology for hourly blood collection over the circadian period and sample processing applied to steers from population A is described in detail by Montanholi et al. [24]. Briefly, the groups of four animals were composed of two efficient and two inefficient steers and offered water and feed for ad libitum consumption over the duration of blood sampling. Steers were blood sampled hourly from noon until 11:00 the following morning. Steers had a heparinized jugular catheter coupled to a tubing placed in between the shoulders to minimize the distress of blood sampling. Blood samples (10 ml) were withdrawn and immediately centrifuged (3000*g* for 25 min at 4 °C), then blood plasma was harvested and stored at −80 °C until further metabolic profile analysis.

### Liver biometrics, sampling and microscopic imaging

Liver weights were measured and reported as whole liver weight and as a proportion of the animal’s live weight. The body weight used for this proportionate trait was the average of the weights measured the day before slaughter and assessed immediately before transportation to the abattoir. The transportation to the abattoir lasted 25 min and steers were slaughtered without prior fasting. The entire liver was removed, inspected and weighed after excising the gallbladder. All the livers sampled were considered healthy by the federal inspector. Liver fragments, sampled within 42 ± 6 min of stunning, were gently collected from the visceral side of the right lobe and adjacent to the insertion of the portal vein. Samples (1.0 × 1.0 × 0.5 cm) were fixed in 10% neutral phosphate buffered formalin for 24 h under gentle agitation and processed for paraffin embedding (Sakura Tissue Tek VIP 6^®^: Sakura Finetek; Alphen aan den Rijn, The Netherlands). Paraffin blocks were sectioned at 5 µm thickness using a microtome (Leica 2255^®^: Leica Biosystems; Wetzlar, Germany). Tissue fragments mounted on glass slides were left to dry overnight on a hot surface (36 °C) and stained with hematoxylin and eosin according to the method described by Carson [25]. Liver tissue slides were first screened by an animal histologist to ensure that all livers were free of any abnormality. Then, liver tissue microscopic images were taken using a Leica DMLB microscope (Leica Microsystems Inc.^®^, Wetzlar, Germany) equipped with a video camera QICAM Fast 1394 (QImaging^®^, Surrey, Canada) connected to the computer-based image analysis software QImaging (QImaging^®^, Surrey, Canada). Images were taken using the portal triad and central vein as histological references [13]. A total of 10 images around each of these two regions were taken from each liver and from a single section at 200× magnification.

### Liver histomorphometry analyses

**Fig. 1:**
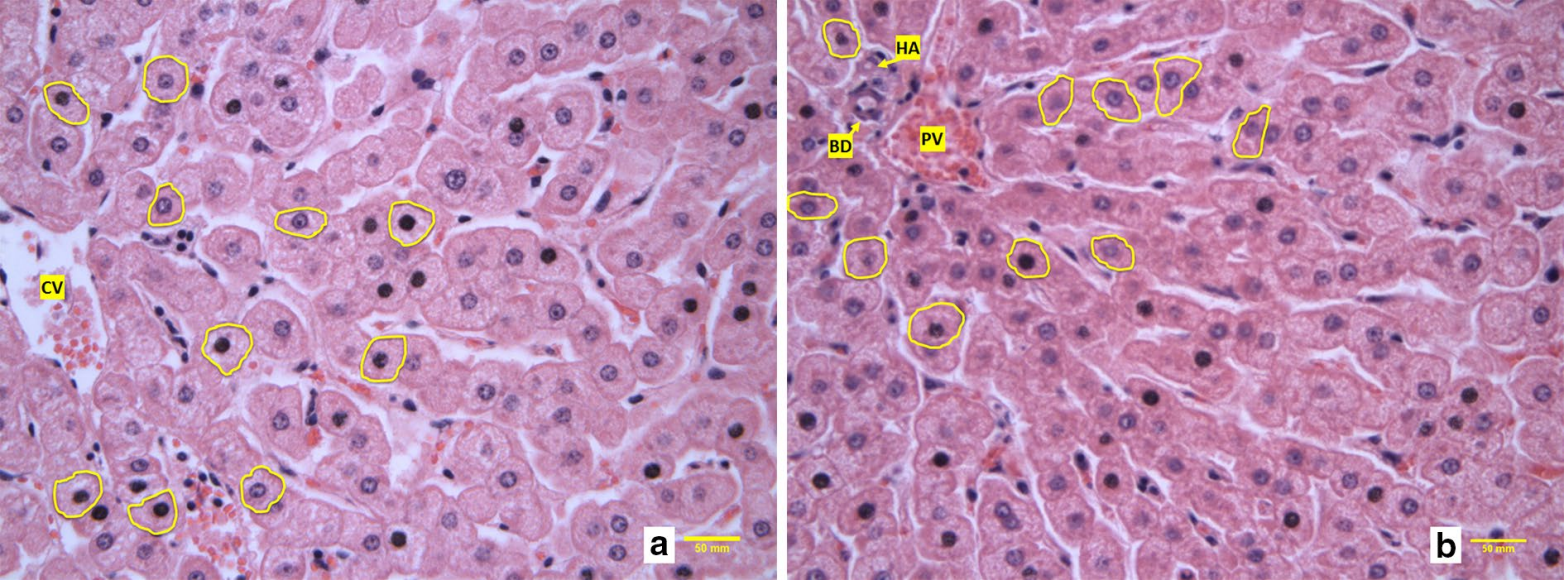
Liver histomorphometry, example of selected hepatocytes around central vein (**a**) and portal triad (**b**) histological regions for measurements on livers from both populations of steers. *CV* central vein, *PV* portal vein, *HA* hepatic artery and, *BD* bile duct

Four measurements performed in both central vein and portal triad regions included the following: hepatocyte area (HA; µm^2^), hepatocyte perimeter (HP; µm), hepatocyte nuclei area (NA; µm^2^) and percentage of the total hepatocyte area occupied by the nuclei (NH; %). Hepatocytes were selected and analyzed by an experienced judge, who was blinded to the feed efficiency group of the corresponding animal. Ten hepatocytes where chosen per image of central vein or portal triad, totaling 100 hepatocytes measured per animal in each histological region. The criteria for selecting hepatocytes were established as the following: proximity to the histological landmark (central vein or portal triad), well delimited cellular boundaries, and presence of round shaped nuclei. The same 100 hepatocytes were used to perform each of the four histological assessments. Histological quantifications were performed using ImageJ^®^ (ImageJ, U. S. National Institutes of Health, Bethesda, USA). Both HA and HP were assessed using a free hand drawing tool by outlining the perimeter of the chosen hepatocytes. This was followed by the application of the threshold option, which highlights the area covered by the nuclei, to determine NA. The NH was calculated as the percentage of the HA occupied by the NA. Figure 1 illustrates the histomorphometric measures (HA, HP and NA).

### Blood plasma metabolic profile

The blood plasma samples were analyzed through an animal diagnostic service (Animal Health Laboratory, University of Guelph, Guelph, Canada). Briefly, an automated analyzer (Cobas^®^ c311/501 analyzer, Roche Diagnostics GmbH, Indianapolis, USA) [26] was used to measure the following blood plasma metabolic parameters: albumin (g/l), aspartate aminotransferase (AST; U/l), γ-glutamyl transpeptidase (GGT; U/l), urea (mmol/l), cholesterol (mmol/l), creatinine (mmol/l), alkaline phosphatase (ALP; U/l), creatine kinase (U/l) and lipase (U/l). Carbon dioxide (CO_2_; mmol/l) levels were determined using an automated analyzer (Cobas^®^ 4000 c311, Roche Diagnostics GmbH, Mannheim, Germany). The determination of β-hydroxybutyrate (µmol/l) was completed using a commercial kit (Randox^®^, RANDOX Laboratories Ltd., Crumlin, UK). Blood plasma acetate (g/l) was determined via spectrophotometry using a commercial kit (K-ACETRM, Megazyme© International, Wicklow, Ireland) according to the manufacturers’ protocol. A colorimetric-based assay was used to determine the concentration of total bile acids (BA; µmol/l) (Diazyme total bile acids, Diazyme©, Poway, USA).

### Statistical analyses

Data were analyzed using the SAS^®^ software (SAS Institute, Cary, USA). Data normality was tested using the univariate procedure for each continuous variable. Non-normal data, based on Anderson–Darling test and kurtosis and skewness out of the −2 and +2 range, were either log or reciprocal transformed and then back-transformed to report the results. Preliminary regression analysis using the general linear model procedure, as was described by Montanholi et al. [3], indicated an absence of significant breed effects (P ≥ 0.10); therefore, breed effect was not included in the analyses detailed below. Additionally, preliminary analysis indicated substantial increase in statistical power by pooling the two populations for the measurements done in common. Thus, means of the two feed efficiency groups for biometry, productive performance and liver morphometry measures were tested using the general linear model procedure and compared using *T* test, according to the following model:

$$Y_{ijk} = \mu + Population_{i} + Efficiency_{j} + \beta (Age_{ijk} - Age..) + \varepsilon_{ijk}$$

where *Y*_*ijk*_ is the dependent variable measured on the *k*th steer, belonging to the *j*th feed efficiency group and sampled in the *i*th population; *µ* is the overall mean effect for the measure; *Population*_*i*_ is the fixed effect of *i*th population (A or B); *Efficiency*_*j*_ is the fixed effect of *j*th feed efficiency group (efficient or inefficient); *β(Age*_*ijk*_–*Age..)* indicates the inclusion of age at slaughter as a covariate; and *ε*_*ijk*_ is the residual random effect associated with the assessment on the *k*th steer. The interaction between population and efficiency group was tested and due to the lack of significance, it was removed from the model described above.

**Fig. 2:**
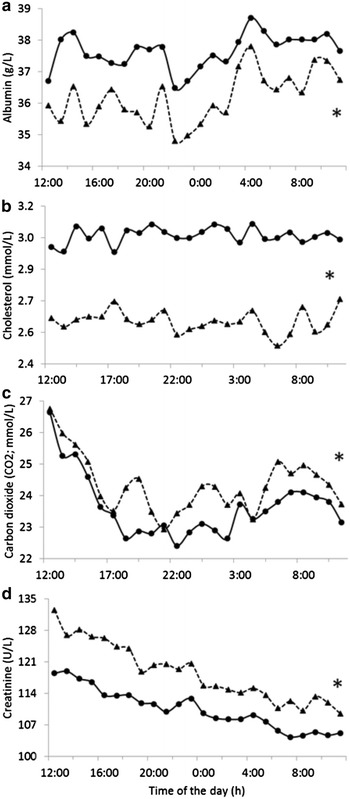
Circadian profile of **a** albumin, **b** cholesterol, **c** carbon dioxide and **d** creatinine. Feed efficient (*filled triangle*) and inefficient (*filled circle*) beef steers. Presence of asterisk denotes P < 0.05 over the circadian period

The repeated measures of blood analytes over the circadian cycle from population A were analyzed through repeated measures using the mixed procedure, according to the following model:

$$Y_{ijk} = \mu + Efficiency_{i} + Time_{ij} + \varepsilon_{ijk}$$

where *Y*_*ijk*_ is the dependent variable, *μ* is the overall mean, *Efficiency*_*i*_ is the fixed effect of feed efficiency groups (*i* = efficient or inefficient), *Time*_*ij*_ is the fixed effect of sampling time within feed efficiency groups (*j* = 1,2,…24), and *ε*_*ijk*_ is the residual random error. Adjustment for age was tested and revealed unnecessary within population A, thus not included in the repeated sampling analysis. Although not included in the model, the interaction between feed efficiency group and time generated the least square means plotted in Fig. 2. The autoregressive covariance structure was selected based upon maximum likelihood according to the Bayesian information criterion and the betwithin degrees of freedom option was used as repeated measures adjustments. The Scheffé’s test was used to compare the least square means of efficient and inefficient steers from the repeated analysis. For all analyses, data were considered statistically significant when P ≤ 0.05 and were considered a trend towards significance when 0.10 ≥ P > 0.05.

## Results

The descriptive statistics and least square mean comparisons of indicators of productive performance are presented in Table 1. The efficient and inefficient groups of steers differed in residual feed intake. Efficient steers consumed 1.85 kg/day less feed (dry matter basis) than inefficient steers while achieving a comparable performance in terms of daily weight gain, body weight and body composition, as measured by ultrasonic assessments of back fat thickness and rib eye area. The biometrics of livers assessed at slaughter are listed in Table 1. No differences were observed between feed efficiency groups for liver weight or liver to body weight ratio.

**Table 1:** Descriptive statistics and least square means of the productivity and liver biometrics by efficiency groups

Measures (unit)	Mean	Standard deviation	Efficient	Inefficient	P value
Residual feed intake (kg/d)	0.00	0.98	−0.90	0.89	0.001*
Average daily gain (kg/d)	1.77	0.25	1.74	1.76	0.662
Average feed intake (kg/d)	9.54	1.22	8.56	10.41	0.001*
Back fat thickness (mm)	12.17	3.06	12.27	12.09	0.885
Rib eye area (cm^2^)	108.77	6.24	109.20	108.41	0.770
Body weight (kg)	535.4	48.86	533.4	545.0	0.361
Liver weight (kg)	7.35	1.09	7.24	7.55	0.363
Liver weight (% body weight)	1.26	0.25	1.26	1.29	0.561

* P ≤ 0.05

Table 2 presents the descriptive statistics and least square mean comparisons of liver histomorphometric measures by histological region (portal triad and central vein). Consistently across the two histological regions, HA, HP and NA were larger in efficient steers. While the relative size of hepatocyte nuclei in relation to hepatocyte size (NH) did not differ between efficiency groups in either of the histological regions. NH was 57.3% larger in the central vein region compared to its size at the portal triad.

**Table 2:** Descriptive statistics and least square means of liver histomorphometry by histological regions and efficiency groups

Region	Measures (abbreviation; unit)	Mean	Standard deviation	Efficient	Inefficient	P value
Portal triad	Hepatocyte area (HA; µm^2^)	302.66	36.29	323.31	286.37	0.001*
	Hepatocyte perimeter (HP; µm)	66.38	5.13	68.55	63.61	0.001*
	Hepatocyte nuclei area (NA; µm^2^)	68.93	14.12	71.73	65.23	0.007*
	Nuclei area by HA (NH; % HA)	4.58	0.78	4.65	4.49	0.232
Central vein	Hepatocyte area (HA; µm^2^)	332.16	38.09	357.43	321.26	0.037*
	Hepatocyte perimeter (HP; µm)	69.23	4.40	70.64	67.76	0.024*
	Hepatocyte nuclei area (NA; µm^2^)	69.61	11.95	71.92	66.93	0.031*
	Nuclei area by HA (NH; % HA)	7.14	1.33	7.10	7.02	0.759

* P ≤ 0.05

The blood analytes were organized in four classes; namely, products related to liver function (albumin, BA, cholesterol and urea), enzymes primarily related to liver function (ALP, AST and GGT) and lipase that is primarily secreted by the pancreatic acinar cells [27] but also described in the liver [28], indicators of energetic status (acetate, BHBA and CO_2_) and indicators of muscle metabolism (CK and creatinine). The descriptive statistics and mean comparisons between feed efficiency groups for metabolic profile parameters are presented in Table 3. Creatinine and CO_2_ levels were greater in blood plasma of efficient steers compared to inefficient steers, while albumin and cholesterol levels were lower in efficient steers. A trend (P ≤ 0.10) was observed suggesting greater levels of lipase in blood of efficient steers. No differences (P ≥ 0.10) between efficiency groups were observed for the remaining analytes evaluated, which include BA, urea, ALP, AST, GGT, acetate, BHBA and CK.

**Table 3:** Descriptive statistics and least square means for metabolic profile parameters by efficiency groups

Parameter (abbreviation; unit)	Mean	Standard deviation	Efficient	Inefficient	P value
Products related to liver function					
Albumin (g/l)	36.92	1.76	36.18	37.65	0.001*
Bile acids (BA; umol/l)	13.75	5.65	9.79	10.64	0.491
Cholesterol (mmol/l)	2.84	0.47	2.62	3.05	0.008*
Urea (mmol/l)	4.66	0.84	4.56	4.49	0.920
Enzymes related to liver function					
Alkaline phosphatase (ALP; U/l)	98.72	18.36	101.80	99.76	0.816
Aspartate aminotransferase (AST; U/l)	103.31	88.97	77.71	79.68	0.830
γ-glutamyl transpeptidase (GGT; U/l)	23.01	4.46	22.69	23.17	0.558
Lipase (U/l)	7.43	0.80	7.71	7.15	0.082**
Indicators of energetic status					
Acetate (µg/ml)	46.33	15.17	47.62	46.13	0.359
β-hydroxybutyrate (BHBA; umol/l)	369.57	84.18	354.15	379.98	0.222
Carbon dioxide (CO_2_; mmol/l)	24.10	1.55	24.36	23.65	0.019*
Indicators of muscle metabolism					
Creatine kinase (CK; U/l)	320.14	180.43	278.57	258.59	0.702
Creatinine (mmol/l)	116.58	15.78	118.59	110.50	0.037*

* P ≤ 0.05

** P ≤ 0.10

The circadian pattern of the blood analytes differing between efficient and inefficient steers are presented in Fig. 2. It is remarkable, the symmetry of the circadian pattern of cattle from distinct feed efficiency groups for albumin and cholesterol levels. It is also noticeable, the similarity of the CO_2_ and creatinine patterns, especially during the first hours of blood sampling.

## Discussion

Steers were monitored for productive performance during the finishing phase of the beef cattle production cycle; a period which is particularly impacted by major expenses associated with the rich diets fed to ensure fast growth and desirable carcass composition [1]. We observed that during this period of 140 days, each of the efficient steers consumed a total of 259 kg less feed (dry matter basis) to achieve similar growth rate, body weight and carcass composition (as indicated by the ultrasonographic assessments of body fatness and leanness) when compared to the inefficient steers. This phenotypic divergence in feed efficiency has also been reported elsewhere [29] and reinforces the economic and environmental [30] benefits of increasing the efficiency of feed utilization in the bovine. Herein, indicators of metabolic rate and liver function are discussed in the context of feed efficiency, not only to advance our knowledge on physiological mechanisms related to energy utilization, but also to identify potential biomarkers that could be applied in the livestock industry to indirectly assess feed efficiency. Such biomarkers could be complementary and of assistance to support molecular approaches of research on gene expression [31] and gene networks [32] as these relate to feed efficiency.

Liver weight appears to increase or decrease in direct proportion to the nutritional plane [33] and across physiological stages [7]. Our results indicated no difference in liver biometrics when efficient and inefficient steers were compared, suggesting that metabolic differences in liver function related to feed efficiency [6] may not be reflected in the liver weight. Gravimetric assessments of organ function are relatively coarse assessments when used to capture diminished structural and functional changes such as those due to variation in feed efficiency. Alternatively, one can assess certain physiological events which result in drastic changes in liver workload and weight. This lack of differences in liver and other visceral organ weights in relation to feed efficiency category is also reported elsewhere [20].

Despite the lack of liver weight differences between feed efficiency groups, the micro-structural evaluation of the liver parenchyma revealed direct associations between hepatocyte and hepatocyte nuclei size with feed efficiency in both histological regions evaluated. Overall, these results indicated that improved feed efficiency is associated with a greater functional workload placed on the liver. It is known that the liver primarily responds to fluctuations in workload through hepatocyte hypertrophy or hypotrophy [7]. For instance, Zaitoun et al. [14] demonstrated that a portacaval shunt resulted in substantial hypotrophy of hepatocytes in rats due to the surgical decrease in liver workload. Compared to other organs that are constantly being renewed (such as the intestine), the hepatic parenchyma is a relatively stable cell population; dividing cells are seldom seen in the normal liver [13]. Thus, the observed enlarged hepatocytes in efficient steers strongly indicates that a higher metabolic pace in liver is consonant with improved feed efficiency. This is further reinforced by the observation of increased mitochondrial enzymatic activity in the liver of cattle with superior feed efficiency [6]. Moreover, the larger hepatocytes found in the efficient steers and the lack of difference in weight when compared to the inefficient steers, suggests an overall reduced number of hepatocytes in the liver of efficient steers, which remains to be evaluated through extensive cellularity studies including other lobes of the liver, combined with cellular turnover assessments [34].

The histomorphometrical findings herein, are also in agreement with observations in the intestine metabolism and structure in the context of feed efficiency and productivity [15], when evaluating the histomorphometry of the small intestine of the steers from population B reported in the present study, found larger cellularity in both the duodenum and ileum, corresponding to a higher functional workload in the small intestine of cattle with superior feed efficiency. Similarly, Steinhoff-Wagner et al. [35] found that breeds of cattle with higher growth rates and superior feed efficiency had greater cellularity in the small intestine compared with the cattle breeds with lower growth rates. Unlike the healthy liver [36], the intestine responds to changes in workload primarily by adjusting its cellularity [11]. In fact, our histomorphometrical results in the liver are in line with findings in the small intestine [15, 35] as both indicate that improved feed efficiency is accompanied by a higher metabolic demand on these visceral organs. Interestingly, a study by Colnot et al. [37] on glucose absorption in the small intestine of neonatal calves demonstrated the primary role of the mucosal growth to trigger the intestinal metabolism of glucose. In another study in calves, Meyer et al. [17] demonstrated the relevance of intestinal growth to the variation in feed efficiency.

Despite the functional differences in hepatocytes neighboring the portal triad and central vein microscopic regions [38], we did not observe histomorphometrical differences between these relating to feed efficiency. However, the relatively larger hepatocyte nuclei area in the central vein region in comparison to the portal triad region is an expected finding [13], and reinforces the soundness of our histomorphometry methodology.

The circadian profile of blood plasma albumin indicated lower concentrations in efficient steers. Gonano et al. [18], also evaluating the circadian cycle, observed no difference in albumin levels according to feed efficiency in beef heifers. Similarly, Bourgon et al. [19] did not find differences on albumin over ultradian sampling in feedlot beef bulls. In another study, Richardson et al. [20] observed a negative correlation between blood plasma albumin and daily feed intake. This observation supports our results, since inefficient steers consumed more feed than efficient steers. Additionally, albumin is solely produced in hepatocytes and is a relatively abundant and large molecule [39] and understanding that the machinery associated with albumin synthesis and secretion represents a large portion of the cytoplasm of hepatocytes [40]. We suggest that the myriad of liver functional differences (i.e., [31, 32]) influencing feed efficiency could be related to the processes involved with production, storage and secretion of albumin by the hepatocytes, which ultimately could influence the histomorphometric differences observed herein. We hypothesize that the secretion of this protein may be enhanced in inefficient animals, resulting in a lower accumulation of albumin in the hepatocytes and partially explaining the diminished cell size in response to lower feed efficiency.

Cholesterol levels exhibited minimal fluctuation throughout the circadian period; this is similar to the findings of Bitman et al. [41] in dairy cows. This relatively stable relationship of cholesterol levels with feed efficiency supports the utilization of cholesterol as a robust indicator of feed efficiency, since fewer blood collections may be sufficient to discriminate groups of cattle by feed efficiency. This result is also supported by the similar findings of Bourgon et al. [19] also studying feedlot cattle. The fact that improved feed efficiency was associated with lower levels of cholesterol, suggests that less lipogenesis, lipid transport and deposition occurs in efficient cattle. The biosynthesis of cholesterol from acetate is an energetically expensive process in the cell [42]; thus, lower cholesterol seems associated with lower basal energy requirements of efficient steers.

These lower levels of cholesterol may also be associated with the suggested increase in levels of lipase in efficient steers. It is known that cholesterol levels are at least partly regulated by pancreatic [43] and hepatic [44] lipases. The hepatic lipase enzyme not only hydrolyzes metabolites in cholesterol, but also stimulates cholesterol ester uptake by hepatocytes [45]. This evidence may also partially explain the enlarged size of the hepatocytes in efficient steers, since liver is the major organ of cholesterol uptake, accounting for 65% of the total [46]. Despite the fact that bile acids are produced in the liver as end products of cholesterol metabolism [47], differences in the size of the cholesterol pool across efficiency groups did not reflect differences in the abundance of bile acids throughout the circadian period; this supports the role of other controlling mechanisms to determine the pool size of bile acids discussed elsewhere [48].

The similarity in the concentration of liver function-related enzymes (ALP, AST and GGT) between efficient and inefficient steers is supported by other studies. Richardson et al. [38] and Bourgon et al. [19] observed no differences for AST and ALP in cattle grouped by feed efficiency. Similarly, Richardson et al. [38] observed no differences for GGT levels in cattle with distinct feed efficiency. On the other hand, Gonano et al. [18] observed increased levels of AST in blood of feed efficient beef heifers. The great variability of these enzymes, potentially due to effects of environment, physiological state and feeding regime, should be addressed in future studies.

Despite the relevance of acetate and BHBA as energy coins in the metabolism of ruminants [42], these parameters did not differ according to feed efficiency classes. Our findings reflect those of Gonano et al. [18], who evaluated acetate over the circadian period for efficient and inefficient beef heifers across different physiological states and noticed no difference between feed efficiency classes, which agrees with the results by Bourgon et al. [19] for acetate. In another study, Richardson et al. [38] found no difference in BHBA between feed efficiency groups of beef steers. The substantial variability of these parameters, even with hourly sampling, is probably the main limitation of the use of these parameters in the characterization of distinct feed efficiency phenotypes.

Conversely, CO_2_ was consistently increased throughout the 24 h of sampling in cattle with improved feed efficiency. Since CO_2_ serves as a fundamental indicator of metabolic rate [49], our results could be interpreted to suggest the increased metabolic rate is associated with improved feed efficiency. However, this is the opposite of the results reported in a study in beef heifers conducted at a comparable physiological stage and age [18]. An explanation for this divergence may relate to the experimental conditions. In our study, steers were sampled in the warmest period of the year in the Northern hemisphere, with an average barn temperature of 24.9 ± 3.2 °C, while the heifers sampled by Gonano et al. [18] were sampled during the winter, with an average barn temperature of 6.2 ± 1.8 °C, which is within the thermal neutral zone for *Bos taurus* [50], unlike our steers. It is known that blood plasma CO_2_ concentration is influenced by heat and cold stress, as evaluated across different breeds of cattle [51]. It is also known that efficient and inefficient cattle differ in their coping styles to stressors, with feed efficient steers being more physiologically capable of dealing with stress [52]. That said, we hypothesize that efficient steers may have a wider range of blood CO_2_ tolerance, which allows these animals to increase their CO_2_ baseline in response to heat stress without relying extensively on the energetically costly dissipation of heat through evaporative heat loss [49]. Contrarily, the less stress-tolerant, inefficient steers will increase their respiration rate at a lower CO_2_ threshold, maintaining their blood CO_2_ at a lower level. Further investigations involving circadian patterns of breathe gasses analysis and blood partial pressure of CO_2_ and oxygen in different environmental conditions are warranted to elucidate this hypothesis.

Creatine kinase is a clinical marker for muscle protein turnover [53] that has been shown to differ according to feed efficiency class. Gonano et al. [18] observed greater levels of CK in efficient heifers at pubertal age. This relationship was inverted when the same heifers were tested over the circadian period during late gestation, which may be related to changes in CK activity in response to aging [54]. In our study, CK did not differ between feed efficiency groups; the same was observed by Richardson et al. [38], when also evaluating beef steers. This enzyme catalyzes the conversion of creatinine to phosphocreatine, a reversible and energetically demanding reaction occurring mostly in skeletal muscle [22]. Conversely, phosphocreatine spontaneously forms phosphocreatine and creatinine under physiological conditions [55]. In our study, levels of creatinine were increased in efficient steers throughout the circadian period. In another study, creatinine was negatively correlated with feed efficiency [38]. Given the dissociation of our results for CK and creatinine, one may suggest that efficient steers may have a larger proportion of the creatinine metabolism occurring without relying on CK, since these animals had a larger creatinine pool in relation to the pool size of CK across the feed efficiency groups.

## Conclusions

Our study provided the first evidence of a relationship between feed efficiency and liver histomorphometry. Improved feed efficiency appears to be associated with enlarged hepatocytes, which may be due to an increased metabolic rate of the liver parenchyma. Further studies utilizing complementary techniques such as micro-calorimetry and molecular biology will provide further advances on this subject. Additionally, the evaluation of the metabolic profile across distinct phenotypes for feed efficiency revealed, that improved feed efficiency is associated with increased metabolism of muscle (higher creatinine) and of the whole body (higher CO_2_ during heat stress) and reduced bloodstream pools of albumin and cholesterol. In essence, these metabolic discrepancies between feed efficient and inefficient cattle may be determinants of the differences in productive performance and potential phenotypes for indirectly assessing feed efficiency upon extensive validation and refinement for field work applications.

## Abbreviations

ALP: alkaline phosphatase
AST: aspartate aminotransferase
BA: bile acids
BKFT: backfat thickness
BW: body weight
BHBA: β-hydroxybutyrate
CK: creatine kinase
CO_2_: carbon dioxide
GGT: γ-glutamyl transpeptidase
HA: hepatocyte area
HP: hepatocyte perimeter
NA: hepatocyte nuclei area
NH: percentage of the total hepatocyte area accounted by the nuclei
RBEA: rib eye area
RFI: residual feed intake
