# Erratum to: Liver morphometrics and metabolic blood profile across divergent phenotypes for feed efficiency in the bovine

**DOI:** 10.1186/s13028-017-0308-x

**Published:** 2017-06-13

**Authors:** Yuri Regis Montanholi, Livia Sadocco Haas, Kendall Carl Swanson, Brenda Lynn Coomber, Shigeto Yamashiro, Stephen Paul Miller

**Affiliations:** 1 Department of Animal Science and Aquaculture, Faculty of Agriculture, Dalhousie University, 58 River Road, Bible Hill, Truro, NS B2N 5E3 Canada; 2 Faculdade de Medicina Veterinária, Universidade Federal do Rio Grande do Sul, Porto Alegre, RS 91540-000 Brazil; 3 Department of Animal Sciences, North Dakota State University, Fargo, ND 58102 USA; 4 Department of Biomedical Sciences, University of Guelph, Guelph, ON N1G 2W1 Canada; 5 Department of Animal Biosciences, University of Guelph, Guelph, ON N1G 2W1 Canada; 6 Angus Genetics Inc, Saint Joseph, MO 64506 USA

## Erratum to: Acta Vet Scand (2017) 59:24 DOI 10.1186/s13028-017-0292-1

After publication of the original article [1] it was brought to our attention that author Brenda Lynn Coomber was incorrectly included as Brenda Lee Coomber. The correct spelling of the name is included in the author list of this erratum and updated in the original article.
